# Eco-Friendly Biosorbents from Biopolymers and Food Waste for Efficient Dye Removal from Wastewater

**DOI:** 10.3390/polym17030291

**Published:** 2025-01-23

**Authors:** Alexandra Cristina Blaga, Ramona Cimpoesu, Ramona-Elena Tataru-Farmus, Daniela Suteu

**Affiliations:** 1 Department of Organic, Biochemical and Food Engineering, “Cristofor Simionescu” Faculty of Chemical Engineering and Environmental Protection, “Gheorghe Asachi” Technical University of Iasi, D. Mangeron Blvd., No. 73, 700050 Iasi, Romania; acblaga@tuiasi.ro; 2 Department of Materials Science, Faculty of Materials Science and Engineering, “Gheorghe Asachi” Technical University of Iasi, D. Mangeron Blvd., No. 41, 700259 Iasi, Romania; ramona.cimpoesu@academic.tuiasi.ro; 3 Department of Chemical Engineering, “Cristofor Simionescu” Faculty of Chemical Engineering and Environmental Protection, “Gheorghe Asachi” Technical University of Iasi, D. Mangeron Blvd., No. 73, 700050 Iasi, Romania; ramona-elena.tataru-farmus@academic.tuiasi.ro

**Keywords:** aqueous solutions, biosorption, chitosan matrix, orange 16, methylene blue, immobilization

## Abstract

Chitosan-based biosorbents are particularly valuable in environmental applications, such as wastewater treatment for contaminant removal. However, several challenges remain in optimizing their production and performance related to improving adsorption efficiency, stability, scalability, cost, and sustainable sourcing for large-scale applications. The removal of Methylene Blue (MB) and Orange 16 (O16) from aqueous solutions was studied using a biosorbent derived from the waste biomass of the brewing industry, specifically *Saccharomyces pastorianus* immobilized into chitosan. The biosorbent (obtained by a straightforward entrapment technique) was characterized using Scanning Electron Microscopy (SEM) and Energy Dispersive X-ray Analysis (EDAX) to evaluate its structural properties. The biosorption behavior toward organic contaminants, specifically a cationic and an anionic dye, was investigated. Key operational factors that influenced the biosorbent’s efficiency were examined, including the initial dye concentration, dye type, pH of the aqueous solution, and the amount of biosorbent used. These factors were evaluated during the initial stage of the biosorption studies to assess their impact on the overall performance and effectiveness of the biosorbent in removing the dyes from aqueous solutions. Using this eco-friendly biosorbent, the biosorption capacities obtained using the Langmuir isotherm model were 212.77 mg/g in the case of MB dye and 285.71 mg/g in the case of O16 mg/g, and the results confirmed that the biosorption process is based on a physical mechanism as suggested by the energy values of the process, E, obtained using the DR model: the obtained values of 6.09 kJ/mol (MB dye) and 7.07 kJ/mol (O16 dye) suggest a process based on electrostatic interaction bonds. These results indicate that residual biomass of *Saccharomyces pastorianus,* as a byproduct of a biotechnological process, can be exploited as a biosorbent by immobilization in an organic matrix (chitosan) for the retention of polluting organic species from the aqueous environment present in aqueous solutions in moderate concentrations.

## 1. Introduction

The growing concern over water pollution, particularly the contamination of wastewater with synthetic dyes, has determined the exploration of sustainable methods for dye removal [1,2]. These dyes, commonly used in the textile, food, and paper industries, are harmful to both the environment and human health. Conventional methods, such as chemical treatments, often involve high costs, chemical waste, and the consumption of valuable resources [2,3,4,5]. One promising approach is biosorption, a process in which diverse and widely available materials (biopolymers and hydrocolloids, microbial and vegetable biomass, and composite materials) are used to adsorb and remove pollutants from aqueous solutions [6,7]. The choice of biosorbent material depends on the type of pollutant, the specific application, and the material’s adsorption efficiency. Because of their special qualities and sustainability, as well as the fact that they can be used as biosorbents to promote waste valorization and environmental sustainability, chitosan, a derivative of chitin, and microbial biomass, or residual yeast, have emerged as attractive options for dye removal from wastewater [7,8,9].

Chitosan is a biopolymer derived from chitin, a natural polymer found in the exoskeletons of crustaceans like shrimp and crabs. It is widely recognized for its excellent biodegradability, non-toxicity, and ability to form films and gels, making it highly effective in wastewater treatment applications. Chitosan possesses abundant functional groups, including amino (-NH_2_) and hydroxyl (-OH) groups, which enable it to interact with a wide range of pollutants, including anionic and cationic dyes [10,11]. These functional groups allow chitosan to form strong bonds with the dye molecules through ionic interactions, electrostatic attractions, and hydrogen bonding. In addition to its high adsorption capacity, chitosan is easy to modify, enhancing its effectiveness as a biosorbent. Chemical modifications, such as cross-linking or grafting with other functional groups, can improve chitosan’s mechanical strength, swelling capacity, and adsorption properties. These modifications make chitosan a versatile and customizable material for dye removal, offering a sustainable alternative to synthetic adsorbents [7,8,9,10,11].

Biosorption has emerged as an alternative wastewater treatment method, offering the potential for wastewater reuse with improved economic and environmental benefits. As a passive, metabolically independent process, it encompasses all interactions between pollutants and biological matrices (biosorbents). Various adsorbents, including agricultural waste, algae, fungi, and bacterial strains, can be utilized for dye adsorption [12]. Residual microbial biomass, which refers to the biomass resulting from industrial fermentation processes, offers a valuable resource for biosorption [13,14,15,16]. Yeast is an abundant byproduct obtained from several fermentation-based industries: brewing, distilling, bioethanol, and winemaking, and it can be easily regenerated for multiple adsorption cycles. Yeast cells, particularly *Saccharomyces* sp., have a high surface area and contain a variety of functional groups, including hydroxyl, carboxyl, and amino groups, which allow for strong interactions with dye molecules. These groups enable yeast to adsorb both cationic and anionic dyes through electrostatic forces, hydrogen bonding, and ion exchange processes [17,18,19]. The use of microbial biomass for biosorption not only provides a sustainable alternative to synthetic adsorbents but also contributes to waste minimization and resource recovery, aligning with the principles of a circular economy [16].

In the last years, depending on a variety of factors, including the chemical properties and structure of the pollutant, the type of biomass (size and structure), pH, temperature, mixing, pollutant concentration, and the quantity of biosorbent, microbial biosorbents derived from bacterial, yeast, or filamentous fungal cells are used for their capacity to bind pollutants (heavy metals, dyes, and antibiotics) through ionic interactions between the functional groups from the polymers contained in the extracellular surface of the dead (inactive) cells and the pollutant [20,21,22,23,24,25]. The combination of chitosan and residual yeast offers a synergistic approach to dye removal. Chitosan can be used as a binding agent to enhance the stability and structural integrity of the yeast biosorbent, improving its adsorption capacity [26]. This hybrid biosorbent system can be designed to separate a wider range of dyes more effectively, with chitosan providing additional functional groups and enhancing the overall adsorption performance of yeast.

Wastewater from various industries—such as textiles, paper manufacturing, and cosmetics—can contain a wide range of dye types, including cationic and anionic dyes. Different types of dyes interact with biosorbents in distinct ways, primarily driven by the charge of both the dye and the biosorbent. Cationic dyes are positively charged and typically interact with biosorbents that have negatively charged functional groups (hydroxyl groups), while anionic dyes are negatively charged and often interact with positively charged functional groups (such as amines or quaternary ammonium groups) [27].

Since different biosorbents may exhibit varying degrees of affinity for cationic versus anionic dyes, using both dye types allows for a more comprehensive evaluation of the biosorbent’s performance [28]. This ensures that the biosorbent is not limited to removing only one type of dye but can handle a wide array of contaminants, making it a more promising candidate for wastewater treatment across different industries.

Methylene Blue (MB) is a water-soluble, cationic dye commonly used in industries such as paper, plastic, textiles, pharmaceuticals, and cosmetics [29]. However, its complex structure makes it non-biodegradable, and at higher concentrations, it becomes toxic and carcinogenic [22]. The presence of MB in wastewater—often highly visible even at concentrations as low as 1 ppm—can result from dyeing processes or the manufacturing of pharmaceuticals and cosmetics. This necessitates the use of efficient, eco-friendly methods for its removal. Traditional treatment options include precipitation, coagulation, photocatalytic degradation, ultrafiltration, nanofiltration, and electrochemical treatments [30,31,32,33].

Another dye considered in this study is Orange 16 (O16)—an anionic, reactive dye used in the textile industry in the finishing stages for dyeing cellulosic or cotton materials/fibers. Orange 16 is a widely used synthetic azo dye, valued for its bright orange color, but it poses significant environmental and health risks due to its persistence and potential toxicity [34]. Azo dyes are known for their complex structures that often resist natural biodegradation, leading to the persistence of these pollutants in the environment. Its removal from wastewater is crucial for maintaining water quality. Like many azo dyes, Orange 16 can be toxic to aquatic life and is a concern for environmental pollution.

Chitosan and its modified forms have been used for dye removal due to their high adsorption capacity. For Methylene Blue, Chatterjee et al. (2011) reported a maximum adsorption capacity, q_max_, of 99 mg/g using chitosan beads, while chitosan hydrogel beads prepared by SDS gelation exhibited an improved q_max_ of 226 mg/g [35]. Tasdelen et al. (2022) achieved a q_max_ of 21 mg/g for chitosan hydrogel and 52.9 mg/g for chitosan/2-acrylamido-2-methylpropane sulfonic acid/kaolinite composite hydrogels [36]. For Orange 16, Wu et al. (2024) obtained q_max_ of 57 mg/g for a cross-linked chitosan-genipin/SiO_2_ adsorbent [37]. Integrating biomass into chitosan-based biocomposites provides a strategic and eco-friendly solution. Kim (2022) analyzed a biocomposite made from chitosan and *E. coli* for Reactive Yellow 2 adsorption from an aqueous solution at pH 4, obtaining a maximum uptake of 679 mg/g, significantly higher than q_max_ 200 mg/g observed for raw *E. coli* biomass [38].

Our previous work investigated the use of a biosorbent derived from *Saccharomyces pastorianus*, immobilized in sodium alginate using two techniques: microencapsulation (with a Buchi microencapsulator) and simple dripping [39]. The biosorbent was evaluated for its ability to remove the organic pollutant Methylene blue, a cationic dye, from aqueous solutions. The results demonstrated that the biosorbent prepared through microencapsulation exhibited the highest sorption capacity, achieving 188.679 mg/g under optimal conditions (pH 9, biosorbent dose of 5.28 g/L, and a contact time of approximately 100 min). Building on these findings, the present study seeks to further explore the potential of *Saccharomyces pastorianus*-based biosorbents for the removal of organic pollutants from aqueous environments.

The biosorbent potential of *Saccharomyces pastorianus,* immobilized using a simple entrapment technique in chitosan, was investigated for the removal of Methylene Blue (MB) and Orange 16 (O16) from aqueous solutions. Using SEM and EDX, the biosorbents were examined, and their effectiveness in the biosorption process was assessed experimentally. By testing both types of dyes, this study can evaluate the biosorbent’s ability to remove a wide range of dye molecules, making it more versatile and applicable to various wastewater treatment scenarios. By identifying the more effective mechanisms under different conditions, this study contributes to a better understanding of the biosorption process, which is essential for optimizing treatment strategies.

## 2. Materials and Methods

### 2.1. Materials

**Figure 1:**
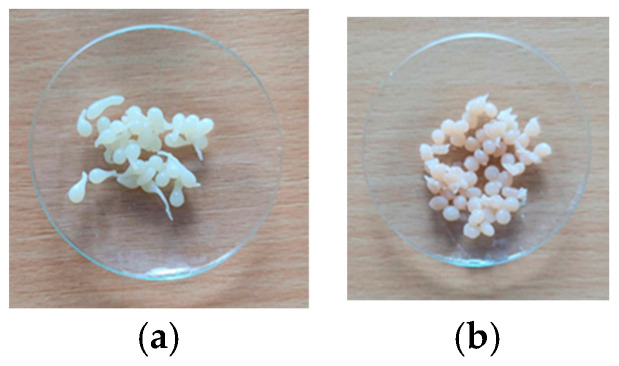
Biosorbents: based on chitosan ((**a**)—CB) and based on *Saccharomyces pastorianus* residual biomass immobilized on chitosan ((**b**)—SpCB).

**Figure 2:**
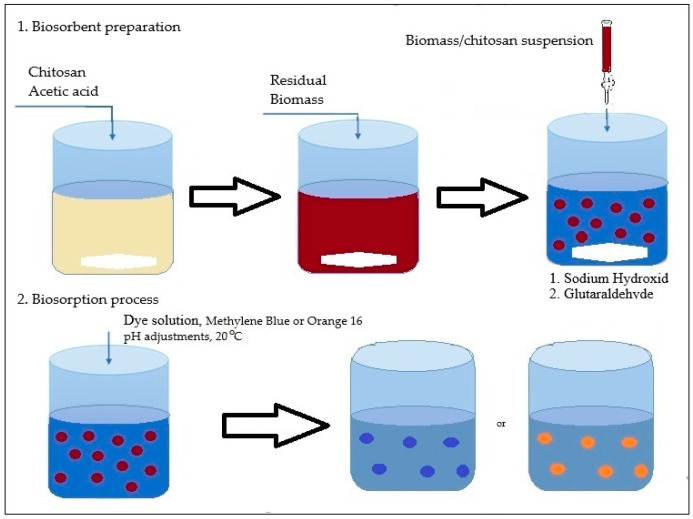
The simple technique procedure for biosorbents preparation SpCB.

In this study, two types of biosorbents were used: based on chitosan (CB) and based on spent biomass immobilized in a chitosan matrix (SpCB) (Figure 1) using a simple dripping technique following previously detailed procedures [39] and systematized in Figure 2.

The industrial residual biomass from *Saccharomyces pastorianus* at Albrau, Onești, Romania, was separated at the end of the fermentation process from the liquid phase by centrifugation at 8000 rpm and dried at 80 °C to break down the cells (rupture of the cells will enhance its binding capacity, providing more functional groups available for adsorption). The dried biomass was subsequently immobilized into chitosan using an immobilization technique that involved the simple dripping of a suspension (5% dry weight residual biomass prepared in a 2.5% chitosan dissolved in 4% acetic acid solution) into a 1% NaOH solution (prepared in distilled water) through a capillary to form ovoidal beads with a diameter of 2 mm. The ovoidal droplets were washed until a neutral pH was achieved and then submerged in a 5% glutaraldehyde solution (a cross-linking agent) to enhance their mechanical properties and stability over a wider pH range.

**Figure 3:**
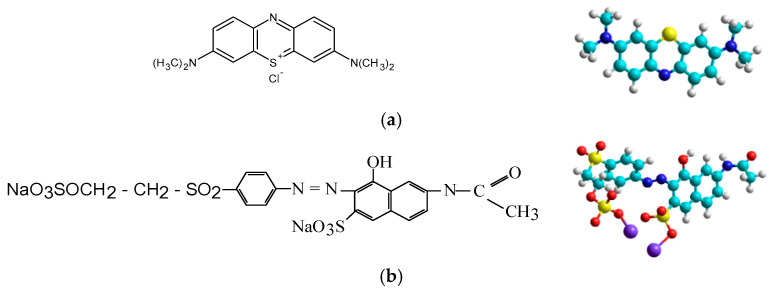
Chemical and molecular structure of cationic dye Methylene Blue (**a**) and anionic reactive dye Orange 16 (**b**).

A cationic dye, Methylene Blue (MB; MW = 319.85 g/mol, λ_max_ = 660 nm, from Merck, Rahway, NJ, USA), and an anionic dye, Orange 16 (O16; MW = 617.54 g/mol, λmax = 495 nm), with the chemical and molecular structure shown in Figure 3, were selected as chemical pollutants of the aqueous system for this study.

Commercial dye powder was used to create the stock solution, which had a concentration of 500 mg MB dye/L and 725 mg O16 dye/L, from which working solutions were made by diluting with distilled water.

### 2.2. Methods

#### 2.2.1. Batch Biosorption Methodology

Experimental biosorption studies were conducted using a straightforward method where biosorbents, were prepared with varying amounts of simple chitosan or chitosan containing 5% d.w. biomass was dried on filter paper and put in contact with 25 mL of dye solutions. The cationic dye solution had initial concentrations ranging from 12.8 to 83.2 mg/L, while the anionic dye solution ranged from 21 to 232 mg/L. The pH was adjusted using 1N HCl or 1N NaOH, and the experiments were carried out at a constant temperature of 20 °C for 20 h. The contact time of the phases was 20 h. The experimental conditions were systematized in Table 1.

The main factors considered in the study were the solution pH, dye type, biosorbent amount, temperature, and initial dye concentration in the aqueous solution (Table 1).

Based on the calibration curve and the Lambert-Beer law, the amount of dye in the solution at equilibrium was measured spectrophotometrically using a Shimadzu UV-1280 UV-VIS spectrophotometer (Shimadzu Corporation, Kyoto, Japan) with maximum dye wavelengths of 660 nm and 495 nm, respectively.

To assess the biosorption capacity (q) of the biosorbents studied in mg dye retained/g biosorbent, the following equation was used:

(1) $$q=\frac{C_{0}-C}{G}\cdot V$$

where C_0_ and C are the initial and equilibrium dye concentrations in the aqueous solution (mg dye/L), G is the amount of biosorbent (CB and SpCB beads) (g), and V is the volume of solution (L).

All biosorption experiments were conducted in duplicates.

#### 2.2.2. Characterization of Biosorbents

The characterization of CB and SpCB biosorbents was made to visualize their internal structure using Scanning Electron Microscopy (SEM).

Scanning Electron Microscopy (SEM) and Energy-dispersive X-ray (EDX) were carried out to characterize the surface micromorphology and structural analysis of the CB and SpCB before and after the biosorption process. A scanning electron microscope, VegaTescan LMH II (Tescan Orsay Holding, Brno—Kohoutovice, Czech Republic), was used. It has a cathode supply voltage of 30 kV, a working distance of 15.5 mm, and a magnification power of up to 60,000×. It is equipped with a secondary electron (SE) detector, a high vacuum, a tungsten filament, and a Vega software (version 3.5.0.0) detector. The details of the chemical composition of the samples were obtained using EDX with an X-ray energy detector (Bruker, X-Flash 6–10, Billerica, MA, USA) connected to the SEM microscope electronic scanning equipment and Esprit 2.1 software, Quantax system—Bruker. Spectra were obtained in automatic BF/ZAF mode and after 12 h maintained in a vacuum.

#### 2.2.3. The Biosorption Equilibrium Data Analysis

The equilibrium data for the biosorption of cationic Methylene Blue and anionic Orange 16 dyes onto two types of biosorbents (CB and SpCB) were analyzed. The maximum biosorption capacity was determined using the Langmuir model [40], which is based on the premise that the maximum biosorption occurs when solute molecules form a monolayer on the biosorbent surface, consisting of a finite number of energetically equivalent sites. The general equation is as follows:

(2) $$q=\frac{K_{L}\cdot C\cdot q_{0}}{1+K_{L}\cdot C}$$

with linearized forms: 1/q = f (1/C)

(3) $$\frac{1}{q}=\frac{1}{q_{0}}+\frac{1}{K_{L}\cdot q_{0}}\cdot \frac{1}{C}$$

where q_0_ and K_L_ are Langmuir constants, q_0_ is the maximum amount of biosorbed solute (mg retained dye/g biosorbent), and K_L_ is the constant related to the solute binding energy (L/mg).

Using the Dubinin—Radushkevich model, we will be able to appreciate the nature of the biosorption process (physical or chemical) depending on the value of the adsorption energy, E, knowing that a value of E < 8 kJ/mol characterizes a physical mechanism of biosorption, while values between 8 and 16 kJ/mol suggest an ion exchange mechanism [40,41]. The general equation is (4) as follows:

(4) $$q=q_{0}\exp\left(-\beta \cdot \varepsilon^{2}\right)$$

with generalized form:

ln q = ln q^0^ − βε^2^ (5)

(6) $$\varepsilon =\mathrm{RT}\;\ln\left(\frac{C+1}{C}\right)$$

(7) $$E=\frac{1}{\sqrt{2\beta }}$$

where q_0_ is the maximum biosorption capacity (mmoli retained dye/g biosorbent); C is the concentration of dye in the solution at equilibrium (mmoli dye/L); ε is the Polanyi potential; β is the activity coefficient related to mean biosorption energy (mol^2^/kJ^2^); E is the mean free energy of biosorption (kJ/mol); R (J/(mol K) is gas constant; T (°K) is absolute temperature.

To obtain the quantitative characteristics of both models, the linearized forms of the general equations were plotted graphically. The values of the characteristic parameters were determined from the intersection with the “y” axis and the slope of the resulting line.

## 3. Results

A comparative study was conducted to evaluate the biosorption capacity of SpCB in comparison to CB in the biosorption process of the selected dyes.

### 3.1. The Biosorbent Physical-Chemical Characterization Before and After Dyes Biosorbtion

**Figure 4:**
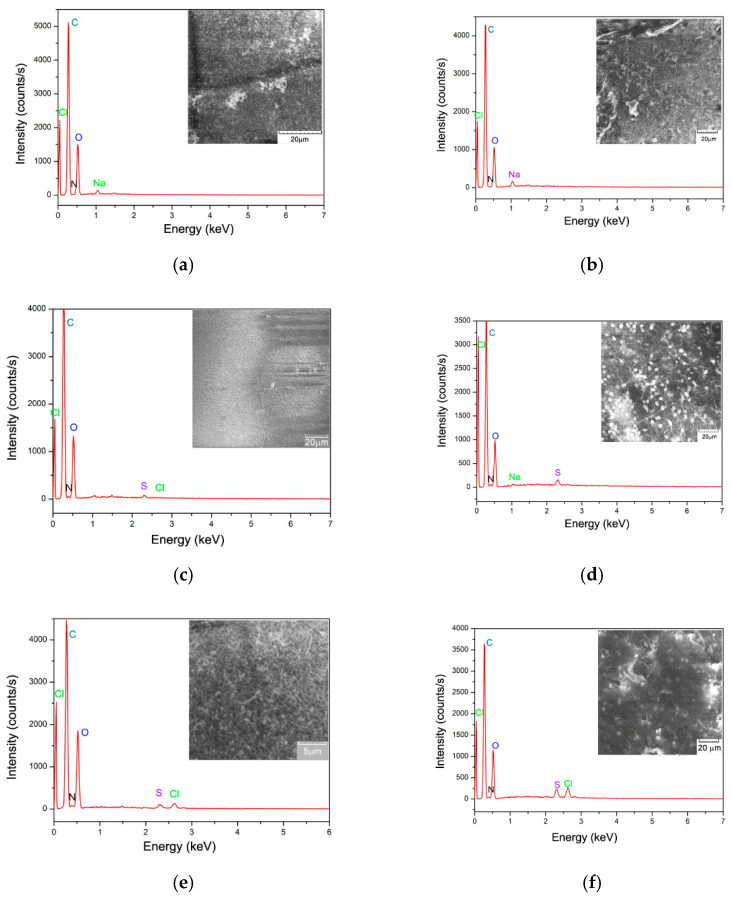
Scanning electron microscopy (SEM) and EDAX spectrum of the obtained polymeric biosorbent, before and after the biosorption process of MB cationic dye and O16 anionic dye: (**a**) chitosan; (**b**) residual biomass of *Saccharomyces pastorianus* immobilized onto the chitosan matrix; (**c**) MB dye retained onto CB; (**d**) MB dye retained on SpCB; (**e**) O16 dye retained onto CB; (**f**) O16 dye retained on SpCB.

Porosity and a large surface area are essential properties of materials used as biosorbents for chemical contaminants. The SEM images presented in Figure 4 highlight the morphology and pore distribution of both CB and SpCB biosorbents before and after dye retention of MB and O16. The SEM analysis reveals that both biosorbents exhibit a mesoporous structure and have a compact, rough surface with high porosity, making them promising candidates for biosorption. Dye molecule retention occurs on the surface of the biosorbent spheres, while their internal structure remains unaffected.

CB and SpCB biosorbents were subjected to elemental analysis and chemical characterization to assess their composition and surface properties. The elemental analysis provided insights into the distribution of key elements, while the chemical characterization helped identify functional groups and surface features critical for biosorption efficiency.

The EDAX spectra of the characterized samples revealed the presence of various elements on the surface, originating from both microbial biomass and chitosan (Figure 4a). Also, the EDX results validate the retention of dyes by the appearance of some chemical elements characteristic of them (such as the presence of Cl^−^ or S^2−^).

### 3.2. Evaluation of the Biosorbent Potential of the Studied Materials

#### 3.2.1. Impact of Key Physico-Chemical Operating Parameters on the Biosorption of Dyes onto Analyzed Biosorbents

**Figure 5:**
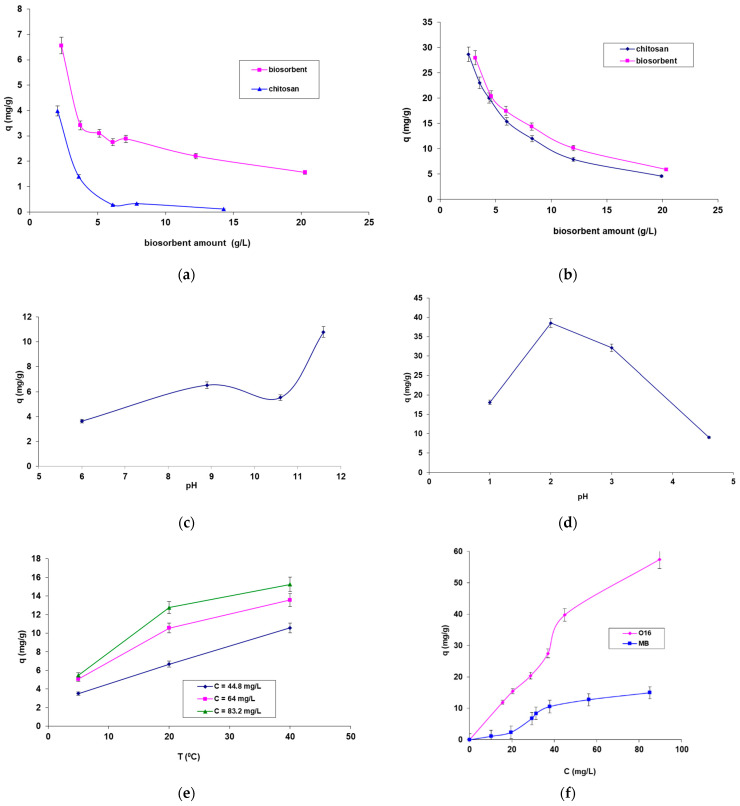
Factors influencing the biosorption of the dyes onto SpCB and CB. The influence of biosorbent dose in the case of biosorbtion of MB (**a**) and O16 (**b**) dye; The influence of pH in the case of MB (**c**) and O16 (**d**); The influence of temperature in the case of MB dye biosorbtion onto biosorbent based on biomass immobilized in chitosan matrix (**e**); The influence of initial concentration of the dyes solution (**f**).

To evaluate the efficiency of biosorbents made from residual biomass immobilized in a chitosan matrix (SpCB), the retention of the cationic Methylene Blue dye and the anionic Orange 16 dye was studied on this material. The results were then compared with those obtained using CB. The study examined the influence of various physico-chemical parameters on the biosorption process for both dyes (Figure 5). Additionally, this investigation aimed to identify the operational parameters that maximize the biosorption capacity.

The examination of the experimental data shown in Figure 5 leads to the following conclusions:

- Figure 5a,b illustrates the influence of biosorbent amount (for both CB and SpCB) on the biosorption of the two selected dyes. The results indicate that both types of biosorbents are capable of adsorbing both dyes, but the biosorption capacity is significantly higher for SpCB. This can be attributed to the cumulative effect of the adsorption capacity of the chitosan matrix and the immobilized residual biomass. Thus, the immobilization of residual biomass allowed bringing it into a form that can be easily manipulated during the technological process but also led to an increased biosorption capacity. Due to these results, the influence of the main factors on biosorption for SpCB was analyzed. When comparing biosorption efficiencies for the two dyes, a higher capacity was observed for the reactive anionic dye O16, likely due to its structure and functional groups, which are more compatible with the surface of the biosorbent. Figure 5a,b show a decrease in the amount of dyes retained per unit mass of biosorbents from 6.559 mg/g to 1.560 mg/g in the case of MB dye onto SpCB (Figure 5a) and, respectively, from 27.956 mg/g to 5.896 mg/g (Figure 4b) in the case of O16 dye onto the same biosorbent as the amount of biosorbent increases from 2.32 g/L to 20.28 g/L.
- The influence of pH (Figure 5c,d) demonstrates that the biosorption of the two dyes is most efficient at distinctly different pH values, depending on the structure of the dye molecules. Specifically, the biosorption of the cationic MB dye on the SpCB-based biosorbent occurs at a strongly basic pH (11.6), while the biosorption of the anionic O16 dye takes place at a strongly acidic pH (2).
- Figure 5e shows that increasing temperature has a positive effect on the biosorption process of the dyes, with MB biosorption on SpCB serving as an example. This variation suggests an endothermic biosorption process. In addition, Figure 5f demonstrates that increasing the initial concentration of the dye causes an increase in the biosorption capacity up to the saturation point of the biosorbent.

Analysis of Figure 5 shows that the biosorption of MB and O16 on CB and SpCB is enhanced by increased temperature and depends on factors such as the type of dye, the amount of biosorbent, and the initial dye concentration. Furthermore, the immobilization of *Saccharomyces pastorianus* biomass in the chitosan matrix not only makes the biosorbent easier to handle during the technological process but also improves dye retention. The chitosan matrix contributes to a slightly higher amount of dye retention, as it possesses inherent sorption properties for these chemical species. In contrast, matrices like sodium alginate, used for immobilizing the same residual biomass, lack these sorption properties [40]. These findings highlight the critical role of pH, biosorbent amount, temperature, and initial dye concentration in optimizing the biosorption process, offering insights for improving the efficiency of dye removal in wastewater treatment applications.

#### 3.2.2. Evaluation of Some Characteristic Quantitative Parameters of the Biosorbtion of the Selected Dyes onto the Studied Biosorbent

**Figure 6:**
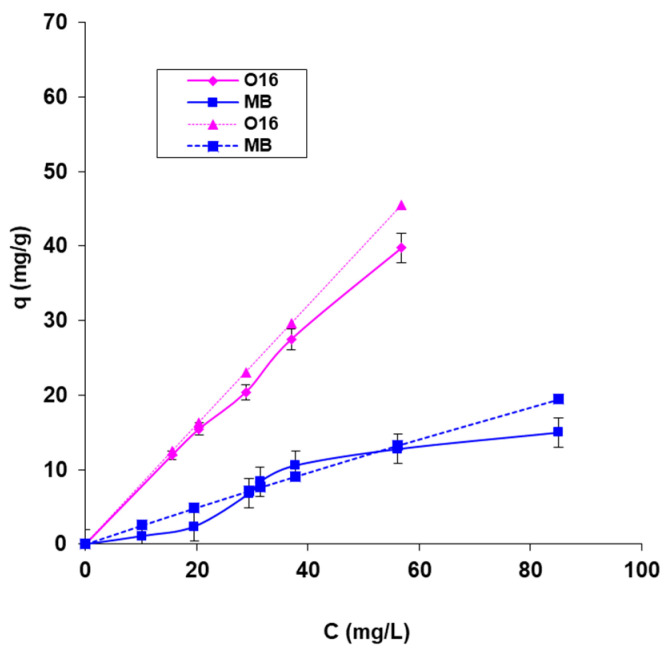
The Langmuir biosorption isotherms for the two dyes, were recorded at 20 °C onto SpCB, experimental (continuous line), and calculated (dotted line).

**Figure 7:**
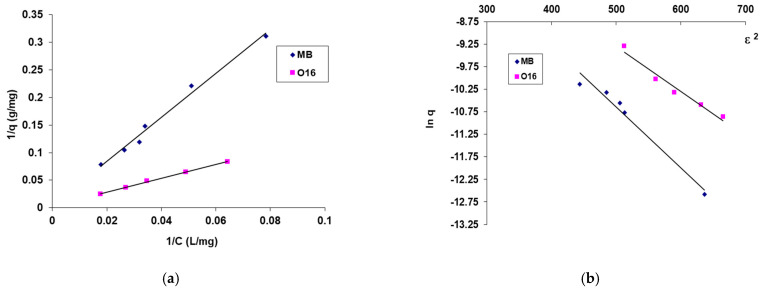
Linearized form of Langmuir (**a**) and DR (**b**) plots for the MB and O16 dye on SpCB. Conditions: (MB) pH = 11.6, contact time = 20 h, biosorbent amount: 2.4 g/L, 20 °C; (O16) pH = 2, contact time = 20 h, biosorbent amount: 2.4 g/L, 20 °C.

The maximum biosorption capacity and biosorption energy, which reveal the nature of the process, were computed using the biosorption isotherms presented in Figure 6 and the graphical depiction of the linearized forms of the adsorption isotherm models that were employed (Langmuir and Dubinin–Radushkevich) (Figure 7). The results are organized in Table 2.

The data in Figure 6 show a good correlation between the isotherms obtained with the calculated data and those obtained with the experimental data, which suggests a good agreement and correctness of the practical experiments.

Studying the data presented in Table 2, the following considerations may be issued:

- It is observed that for SpCB, the maximum biosorption capacity for the reactive dye O16 (285.71 mg/g) is significantly higher than for the cationic dye MB (212.77 mg/g). These findings support previous results that the higher biosorption capacity for O16 is due to a stronger affinity between the functional groups of the anionic dye molecule (SO_3_^2−^) and the functional groups on the surface of the biosorbent, derived from both the chitosan matrix and the immobilized microbial biomass.
- The mean free biosorption energy, E, calculated by the DR equation, can be useful to estimate the nature of the biosorption process (physical or chemical) [41]. In this case, the energy value, E, is 6.086 kJ/mol in the case of MB dye biosorption and 7.071 kJ/mol in the case of O16 dye biosorption, suggesting for the studied dyes biosorption, a physical mechanism as a result of the electrostatic interaction bonds (the sorption energy is less than 8 kJ/mol) [40,41].
- If we take into account the superior value of the maximum absorption capacity presented in Table 2 in the case of the MB dye obtained in the case of SpCB (212 mg/g) and the one from the previous study [39] performed on biosorbents based on the same biomass of *Saccharomyces pastorianus* immobilized in a sodium alginate matrix by a simple entrapment technique (40.8 mg/g) and by microencapsulation with the Buchi equipment (200 mg/g), current results prove the fact that the biosorbent obtained by immobilization of this biomass in a chitosan matrix (even in the simple version of immobilization) is much more efficient. This fact can be attributed to the biosorption capacity of the matrix itself.
- Similar results have been reported in the literature, such as in the biosorption of Orange II and Indigo Carmine dyes on biosorbents made by immobilizing *Saccharomyces pastorianus* biomass in the sodium alginate and chitosan matrices. The dye retention for the alginate matrix was 27.8% and 58.2%, respectively, while for the chitosan matrix, it was 40.8% and 77.9%, respectively [42]. Additionally, studies by Kim S. demonstrate that using chitosan as an immobilization matrix for industrial fermentation waste biomass of *Escherichia coli* in biosorbents for retaining the reactive dye Reactive Yellow 2 from an aqueous medium resulted in a retention capacity of 679 ± 23 mg/g [38]. Compared to studies realized using chitosan or modified chitosan [35,36,37] and the results presented in Table 3, the obtained results showed superior values for adsorption capacity.

All the information leads to the idea that the biosorbent obtained by immobilizing the residual biomass of *Saccharomyces pastorianus* in the chitosan matrix has a high capacity to retain dyes (to the tested capacity of the biomass, that of the chitosan is also added), its value depending mainly on the type of dye (size and functional groups) but also the physical factors that ensure optimal conditions for the biosorption process.

## 4. Conclusions

Sustainable dye removal from wastewater is an essential challenge in mitigating environmental pollution. The use of biosorbents derived from chitosan and residual yeast represents a promising and environmentally friendly solution. These materials are abundant, biodegradable, and capable of efficiently removing both cationic and anionic dyes from wastewater. Moreover, using both materials together offers an environmentally friendly solution that promotes circular economy principles. Chitosan, derived from renewable marine sources, and residual yeast, a by-product of industrial processes, contribute to waste reduction and resource recovery. The use of both cationic and anionic dyes in a biosorption study provides a comprehensive assessment of the biosorbent’s ability to tackle the diverse and complex nature of dye pollution in wastewater.

The Langmuir isotherm model was used to determine the maximum biosorption capacity as 212.77 mg/g for MB dye and 285.71 mg/g for O16 dye on SpCB, with values varying depending on the dye type and experimental parameters. Additionally, the biosorption process, indicated by the energy values (E) obtained from the DR model, suggests a physical mechanism driven by electrostatic interactions.

These results indicate that microbial biomass of *Saccharomyces pastorianus* as a byproduct of a biotechnological process can be exploited as a biosorbent by immobilization in an organic matrix (chitosan in this case) for the retention of polluting organic species from the aqueous environment present in aqueous solutions in moderate concentrations.

## Figures and Tables

**Table 1:** Physical-chemical parameters that influence the biosorption of Methylene Blue and Orange 16 dyes onto microbial biosorbent.

Parameters	Studied Limits of Variation	
	MB	O-16
pH	6–11	1–7
Biosorbent dose (g/L)	0.2–2.028	0.256–1.992
Initial dye concentration in solution (mg/L)	12.8–83.2	21–232
Temperature (°C)	5; 20; 40	

**Table 2:** Characteristic parameters for the biosorption of MB and O16 on SpCB.

Isotherm	Dyes	
	MB	O16
**Langmuir**		
q_0_ (mg/g)	212.77	285.71
q_0_ (mmol/g)	20.26	13.41
K_L_ (L/g)	0.0012	0.0028
R^2^	0.988	0.997
**Dubinin–Radushkevich (DR)**		
q_0_ (mg/g)	6478.9	8280.8
β (mol^2^/kJ^2^)	0.0135	0.01
E (kJ/mol)	6.09	7.07
R^2^	0.969	0.962

**Table 3:** Chitosan or modified chitosan used as biosorbent for MB and O16 dyes biosorption [12,43,44,45].

No.	Dye	Biosorbent	q_max_ (mg/g)
1	Methylene blue	Millimeter-sized chitosan/carboxymethyl cellulose hollow capsule	64.6
2		*Sargassum dentifolium*	66.6
3		*Pseudomonas aeruginosa* USM-AR2/SiO_2_	75.7
4		*Chlorella pyrenoidosa*	212
5		*Aspergillus carbonarius*	21.88
6	Orange 16	*Psyllium* seed powder	100
7		*Labeo rohita*	114.2
8		Pine shell-char	314
9		*Corynebacterium glutamicum*	156.6

## Data Availability

The data presented in this study are available on request from the corresponding author.
